# Acute macular neuroretinopathy in a patient with acute myeloid leukemia and deceased by COVID-19: a case report

**DOI:** 10.1186/s12348-020-00231-1

**Published:** 2021-01-08

**Authors:** Ghodsieh Zamani, Sajjad Ataei Azimi;, Ali Aminizadeh, Elham Shams Abadi, Mostafa Kamandi, Hasan Mortazi, Somayeh Shariat, Mojtaba Abrishami

**Affiliations:** 1 Eye Research Center, Mashhad University of Medical Sciences, Mashhad, Iran; 2 Department of Hematology-Oncology, Faculty of Medicine, Mashhad University of Medical Sciences, Mashhad, Iran; 3 Eye Research Center, Khatam-al-Anbia Eye Hospital, Qarani Blvd, Mashhad, 9195965919 Iran

**Keywords:** Acute myeloid leukemia, Acute macular Neuroretinopathy, Severe acute respiratory syndrome Coronavirus-2, Coronavirus disease 2019

## Abstract

**Purpose:**

Acute macular neuroretinopathy (AMN) is a visual-deteriorating rare clinical entity with an uncertain etiology. We aimed to report a case of AMN and underlying disease of acute myeloid leukemia (AML).

**Case presentation:**

A thirty-five-year-old female patient with bone marrow biopsy confirmed AML, and bicytopenia, under chemotherapy, complained of sudden paracentral visual field defect in her right eye was referred. Visual acuity was 20/20 in both eyes. Posterior segment evaluation revealed multiple Roth’s spots. Optical coherence tomography (OCT) demonstrated hyper-reflectivity band, in the outer nuclear layer and outer plexiform layer, nasal to the fovea of the right eye, and hyperreflective patch in outer retina segmentation en-face OCT, suggestive of the diagnosis of AMN. Nine days after AMN diagnosis, dyspnea, malaise, and cough was initiated. Ground glass opacities in lung CT scan, beside reverse transcription polymerase chain reaction of severe acute respiratory syndrome coronavirus-2, was conclusive of coronavirus disease 2019 (COVID-19). The patient deceased after 6 days.

**Conclusion:**

We report a rare case of AMN following AML. Our findings support the role of ischemia in the outer retina, of which AML may contributed to the pathophysiological process. The patient has deceased less than 2 weeks from AMN initiation.

## Background

Acute myeloid leukemia (AML) is the most common leukemia in adults. Ophthalmic findings are common in patients with leukemia and have been described in near to 90% of the patients [1]. Patients may develop Roth’s spots, retinal hemorrhages in different levels, vascular sheathing, cotton-wool spots, and vascular changes like dilation and tortuosity of the retinal veins.

Acute macular neuroretinopathy (AMN), a rare retinal disorder, is an acutely visual decreasing macular lesion described as a wedge-shaped reddish-brown lesion directed toward the fovea, corresponding with paracentral scotoma, usually in young female patients [2]. In spectral-domain optical coherence tomography (OCT), hyperreflective bands in outer retina, more precisely, hyper-reflectivity of the outer plexiform layer (OPL), and outer nuclear layer (ONL) are found, which ultimately lead to localized thinning of the ONL [2]. The etiologies described are usually associated with a vascular theory, based on the vasoactive nature of the pathology. Recently, in a case series, associations between AMN and leukemia and many other diseases was reported, all have anemia and thrombocytopenia in common [3].

Coronavirus disease 2019 (COVID-19) was primarily identified with severe acute respiratory syndrome, but body hyperinflammatory response, coupled with many organs presenting angiotensin-converting enzyme (ACE) 2, the main receptor of the virus, has been associated with multi-organ complications of the disease [4]. Primarily, ocular manifestations were defined as ocular external diseases like conjunctivitis [5]. Recently, possibly of retinal involvement is reported [6] and also, virus particles were found in autopsy samples of retina [7].

In this report, we aimed to report a case of young female patient with AML, presented with AMN, who has involved with COVID-19, less than 2 weeks after AMN presentation.

## Case presentation

**Fig. 1:**
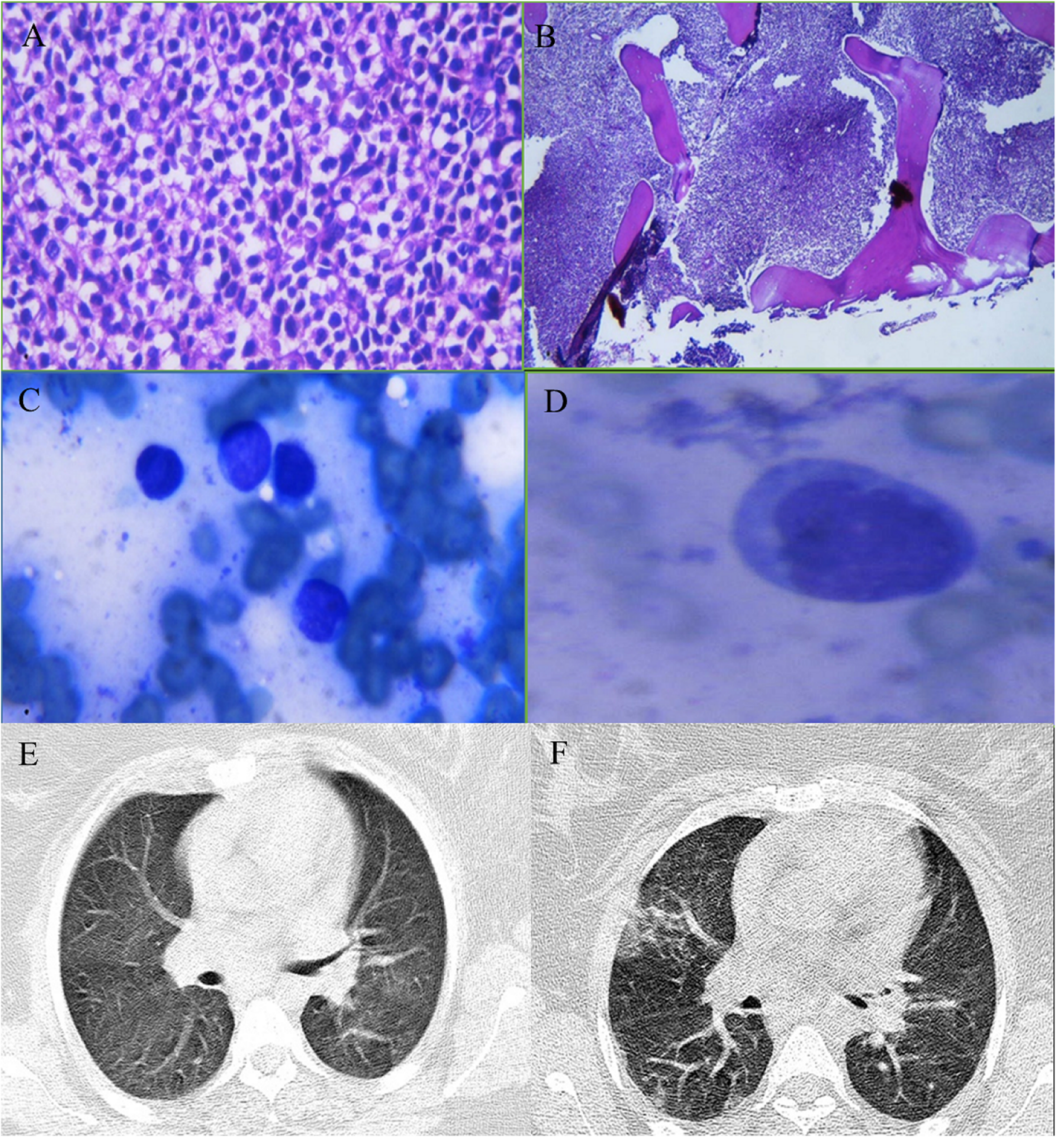
**a**, **b** Bone marrow biopsy result compatible with AML showing a hypercellular marrow with excess of immature myeloid cells. **c**, **d** Bone marrow aspiration shows blast cells are large in size and had a high nucleus/cytoplasm ratio, monocytoid aspect, and blue-gray cytoplasm. **e** Chest CT-scan at the admission, normal without opacities. **f** Chest CT- scan after COVID-19 involvement

A 35-year-old woman presented with history of weakness, metrorrhagia and exertional dyspnea was referred to hematology department. Lab tests revealed leukocytosis (27.7 × 10^9^ /L), anemia (hemoglobin:9.2 g/dl), and thrombocytopenia (56 × 10^9^/L). Peripheral blood smear showed abnormal monocytic cells with significant number of blast (50%) that suggestive of acute leukemia. A diagnosis of AML-M4 was made based on bone marrow aspiration, as acute leukemia with differentiation along both myeloid and monocytic lines were observed (Fig. 1) and also flow-cytometry result. The chemotherapy consisting of Cytarabine and Daunorubicin was initiated immediately after the diagnosis.

**Fig. 2:**
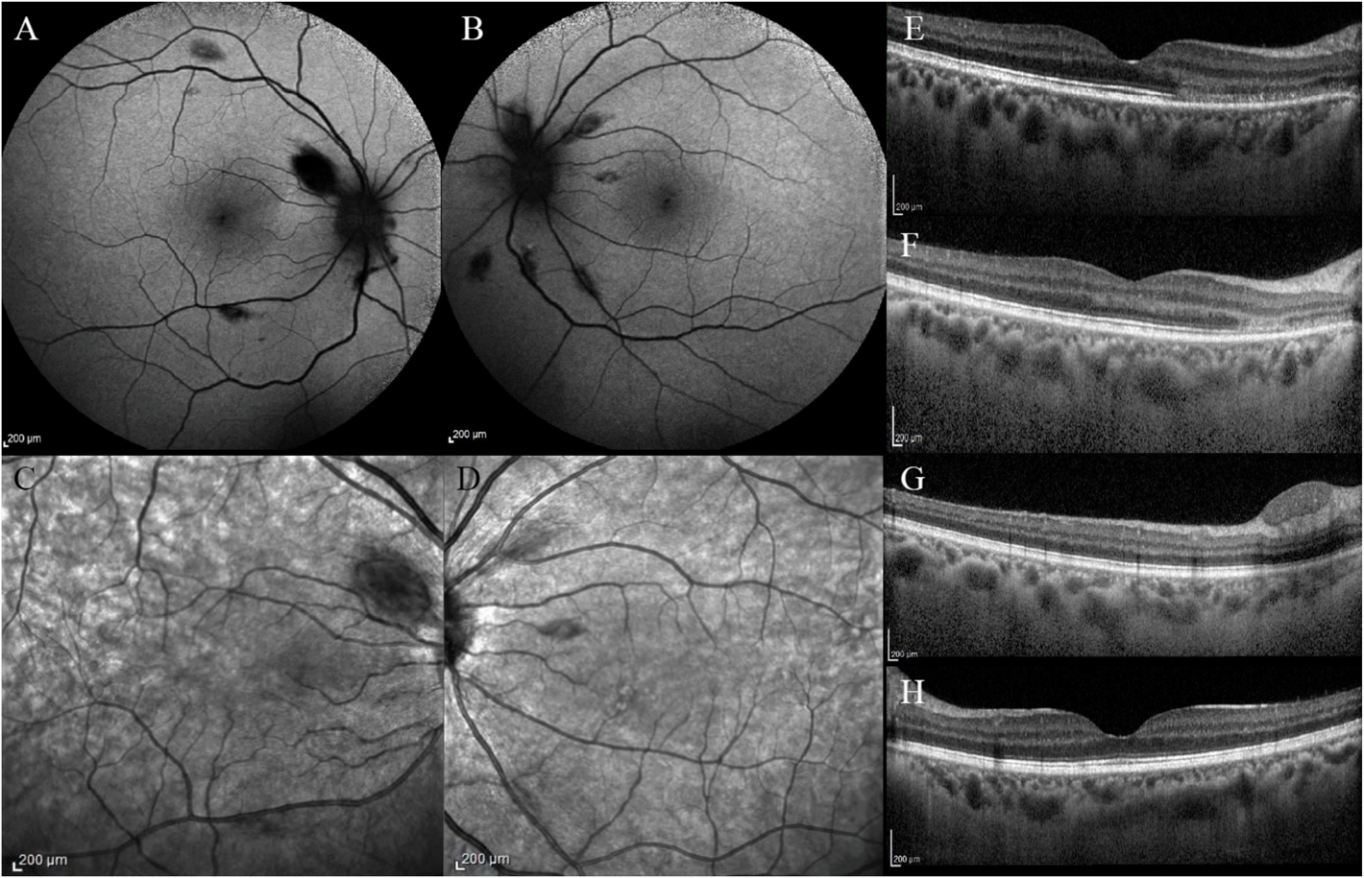
**a**, **b**: Blue autofluorescence imaging shows multiple hypoautofluorescence lesions in both eyes, corresponding intraretinal hemorrhages and Roth’s spots; and normal hypoautofluorescence of the fovea. Small mild hyperautofluorescence area in the papilomacular bundle area is seen, corresponding to the area with acute macular neuroretinopathy involvement, and blockage of the normal autofluorescence of the underlying RPE. **c**, **d**: Near infra-red reflectance imaging showing hyporeflective lesion in the papillomacular area of the right eye and multiple drop like hyporeflective areas associated with Roths spots. **e**, **f** Spectral domain-optical coherence tomography (OCT) demonstrated hyper-reflectivity of the outer nuclear layer (ONL) and outer plexiform layer (OPL) associated with attenuation of the ellipsoid zone (EZ) nasal to the fovea of the right eye. **g** OCT demonstrated epiretinal hemorrhage of the right eye, corresponding to the clinical Roth’s spot. **h** Normal macular OCT of the left eye

**Fig. 3:**
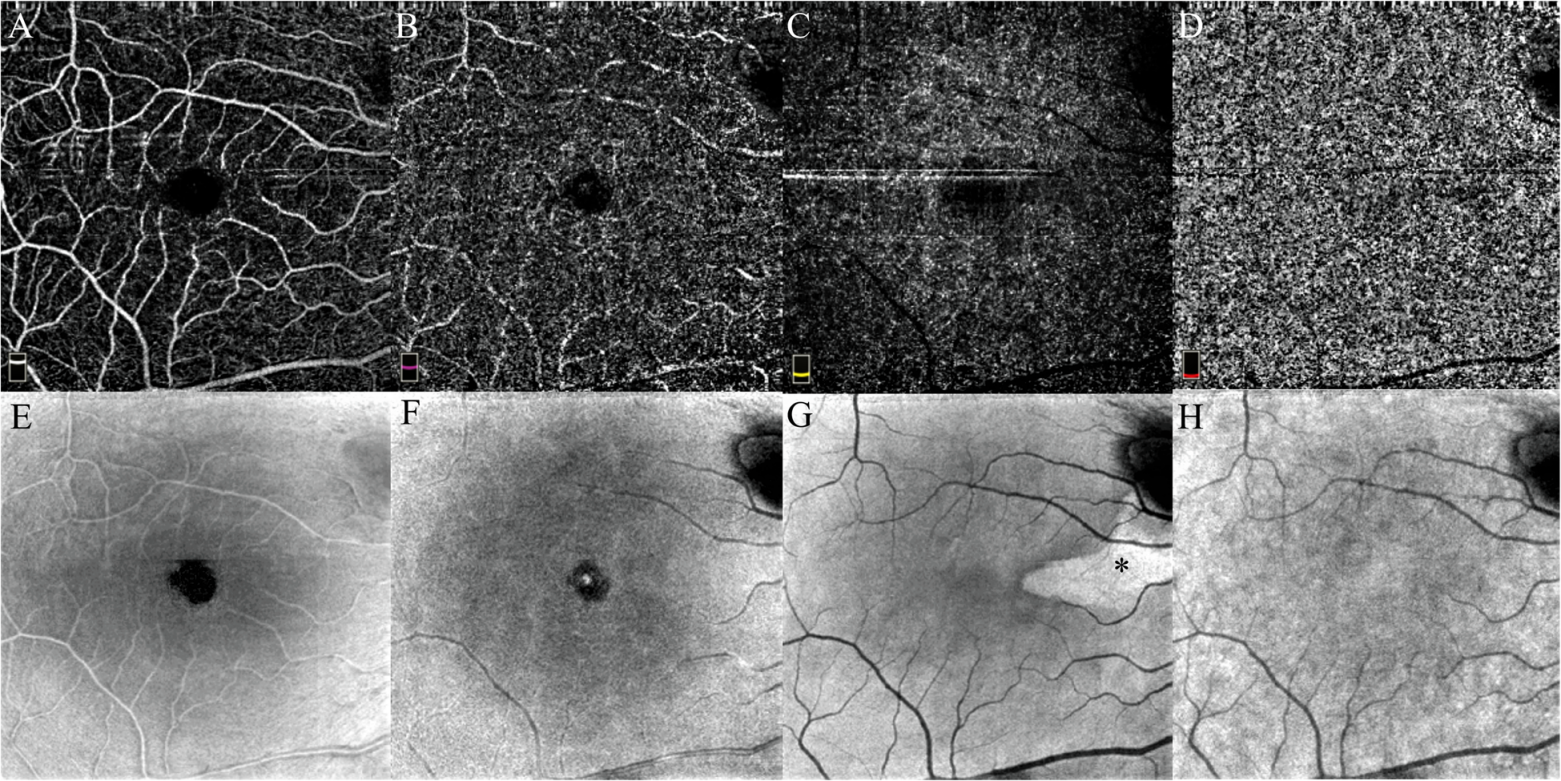
Spectral domain 6 × 6 mm optical coherence tomography angiography (OCTA) (**a**-**d**) and en-face optical coherence tomography (OCT) (**e**-**h**) scan of the right eye macula, at superficial retina (**a**, **e**) deep retina (**b**, **f**), outer retina (**c**, **g**), and choriocapillaris (**d**, **h**) slab selection. At the outer retina slab selection, en-face OCT showed hyperreflective patch (*), corresponding to the edema of the outer retina at the acute macular neuroretinopathy involvement

Five days after initiation of chemotherapy, she also complained of sudden painless paracentral visual field defect and photopsia in her right eye. Visual acuity was 20/20 in both eyes. Intraocular pressure was within normal limits. Slit lamp biomicroscopy of the anterior segment was unremarkable. Fundus findings revealed multiple hemorrhages with white or pale center (Roth’s spots) around the optic disk and vascular arcades in both eyes. OCT demonstrated hyper-reflectivity of the outer nuclear layer (ONL) and outer plexiform layer (OPL) associated with attenuation of the ellipsoid zone (EZ) nasal to the fovea of the right eye (Fig. 2). Outer retina segmentation en-face OCT revealed hyperreflective patch (Fig. 3). These findings did not involve the fovea. They were compatible with the diagnosis of AMN.

Nine days after AMN diagnosis, dyspnea, malaise, and cough was gradually initiated. Although the lungs were clear in the CT scan performed at the admission, the CT scan after deterioration of the systemic status of the patient showed multilobar peripheral ground glass haziness and patchy consolidation foci (Fig. 1). Based on the positive result of nasopharyngeal reverse transcription polymerase chain reaction (RT-PCR) for severe acute respiratory syndrome coronavirus-2 (SARS-CoV-2) and leukopenia (0.5 × 10^9^ /L), the diagnosis of coronavirus disease 2019 (COVID-19) was confirmed. The patient deceased after 6 days because of severe pneumonia.

## Discussion

In this case report, the diagnosis of AMN was confirmed with multimodal imaging techniques in a patient with AML. We found outer retinal changes, like OPL and ONL hyperreflective bands, EZ disruption and en-face OCT hyperreflective patch. Moreover, multiple Roth’s spots were also found in fundus exam, hyperreflective elevations on the inner side of nerve fiber layer.

AMN is a rare retinal disorder which the pathogenesis remains unknown. A vascular theory has been proposed, based on the nature of the precipitating factors as well as the anatomical localization of pathology. It is currently thought to be a result of ischemia of the deep capillary plexuses and may theoretically be seen in any patient with retinal vascular disease or systemic vasculopathic risk factors [3].

As yet, numerous underlying diseases have been reported in association with this disease including viral and flu-like illnesses and vaccination [2, 3, 8, 9] and diseases have anemia and thrombocytopenia in common like leukemia, dengue fever, ulcerative colitis and chronic kidney disease [3].

In the ocular complications of leukemia, profound anemia and thrombocytopenia may cause retinal capillary ischemia due to lack of oxygen supply, or due to endothelial injury at the superficial (cotton wool spot) and intermediate and deep levels, leading to manifestation of paracentral acute middle maculopathy or AMN, respectively [1, 3]. The case presented here highlight thrombocytopenia, anemia and leukocytosis as potential factors contributing to the onset of AMN. Multiple mechanisms presumably may lead to ischemia of the deep capillary plexuses and the development of AMN. Such mechanisms include: reduced blood flow and ischemia due to leukostasis, hyperviscosity syndrome, leukoembolization, endothelial lesion, localized thrombosis secondary to toxic products released by the leukemic cells, and anemia.

ACE2, an essential cell membranes enzyme, is found as the main receptor for SARS-CoV-2. Beside lung type II alveolar cells, ACE2 is mainly found in arterial and venous endothelial cells and arterial smooth muscle cells in most organs [8]. Although ACE2 has been found in human retina, ACE, the hologous enzyme of ACE2, has been reported to present in choroid and different cell types of retina, such as Muller cells and retinal vessel endothelial cells. On the other hand, acute influenza virus infection and administration of an influenza vaccination have been reported as underlying diseases in association with AMN [9, 10]. Hence, it seems that is not too far to associate AMN to SARS-CoV-2, as viral infections have been reported in associated with AMN and the virus itself associated with retinal vascular structure involvement. Recently, Virgo and Mohamed in two cases reported PAMM and AMN in recovered COVID-19 patients [11]. In another report, a patient with visual loss during acute SARS-CoV-2 infection was found to have Roth’s spot, and both AMN and PAMM lesions [12]. This patient was very similar to our patient, as he had also Roth’s spots beside AMN. In a case-control study, in macular OCTA analysis of COVID-19 patients, vascular density of superficial and deep vascular plexuses was lower than normal controls in fovea and parafoveal regions [13]. In other organs, COVID-19 was also associated with ischemic findings. In a case report, large-vessel stroke was considered as a presenting feature of COVID-19 in a young patient, as coagulopathy and vascular endothelial dysfunction have been proposed as complications of COVID-19 [14]. Moreover, in a series of twenty cases with acute limb injuries who suffered from COVID-19, it was proposed that COVID-19 infection might increase the incidence of ALI and be associated with poorer surgical results because of associated acquired hypercoagulability [15].

In conclusion, we have reported a young female patient under chemotherapy for AML-M4 presented with scotoma in her right eye, with hyperreflective bands in outer retina in SD-OCT, who diagnosed as AMN. The patient was deceased because of COVID-19, a couple of days after AMN presentation. In our patient, although AMN findings are mostly attributable to AML, it is unclear the association of AMN and COVID-19 infection in this patient. Although SARS—CoV-2 infection has proposed to be associated, in our patient we don’t know whether the infection predisposed the patient to AMN or the AMN is just associated to AML.

## Data Availability

The datasets used and/or analyzed during the current study are available from the corresponding author on reasonable request.

## Abbreviations

AMN: Acute macular neuroretinopathy
AML: Acute myeloid leukemia
AML-M4: Acute myeloid leukemia type M4
OPL: Outer plexiform layer
ONL: Outer nuclear layer
RT-PCR: Reverse transcription polymerase chain reaction
SARS-CoV-2: Severe acute respiratory syndrome coronavirus-2
COVID-19: Coronavirus disease 2019
OCT: Optical coherent tomography
EZ: Ellipsoid zone
